# Fog Computing for Control of Cyber-Physical Systems in Industry Using BCI

**DOI:** 10.3390/s24010149

**Published:** 2023-12-27

**Authors:** Paula Ivone Rodríguez-Azar, Jose Manuel Mejía-Muñoz, Oliverio Cruz-Mejía, Rafael Torres-Escobar, Lucero Verónica Ruelas López

**Affiliations:** 1 Departamento de Ingeniería Industrial y Manufactura, Instituto de Ingeniería y Tecnología, Universidad Autónoma de Ciudad Juárez, Ciudad Juárez 32310, Mexico; 2 Departamento de Ingeniería Eléctrica, Instituto de Ingenieria y Tecnologia, Universidad Autónoma de Ciudad Juárez, Ciudad Juárez 32310, Mexico; al168396@alumnos.uacj.mx; 3 Departamento de Ingeniería Industrial, FES Aragón, Universidad Nacional Autónoma de México, Mexico 57171, Mexico; oliverio.cruz.mejia@comunidad.unam.mx; 4 Facultad de Ingeneria, Universidad Anáhuac México, Mexico 52786, Mexico; rafael.torrese@anahuac.mx

**Keywords:** fog computing, BCI, machine learning, random forest

## Abstract

Brain-computer interfaces use signals from the brain, such as EEG, to determine brain states, which in turn can be used to issue commands, for example, to control industrial machinery. While Cloud computing can aid in the creation and operation of industrial multi-user BCI systems, the vast amount of data generated from EEG signals can lead to slow response time and bandwidth problems. Fog computing reduces latency in high-demand computation networks. Hence, this paper introduces a fog computing solution for BCI processing. The solution consists in using fog nodes that incorporate machine learning algorithms to convert EEG signals into commands to control a cyber-physical system. The machine learning module uses a deep learning encoder to generate feature images from EEG signals that are subsequently classified into commands by a random forest. The classification scheme is compared using various classifiers, being the random forest the one that obtained the best performance. Additionally, a comparison was made between the fog computing approach and using only cloud computing through the use of a fog computing simulator. The results indicate that the fog computing method resulted in less latency compared to the solely cloud computing approach.

## 1. Introduction

Brain-Computer Interface (BCI) is a technology emerging in recent decades, whose aim is to provide a direct interface with the human brain as an alternative to body-mediated communication.

Thus a BCI system offers a way to control systems or devices without involving any muscle movements [1], which could result in systems that are friendly to users with some physical incapacity. As [2] mentions, BCI technology is helpful for patients with severe brain nerve damage, because in these cases the normal communication channel has been damaged. Also in [1] is commented that persons suffering neuromuscular diseases, can use BCI to perform multiple tasks such as controlling a machine or accessing communication devices, in [3,4,5] this list is extended to manipulators, exoskeleton control, robotics, electric wheelchair control, text input, intelligent home control, and industrial equipment. In this paper, we are interested in this last activity.

Recently, the potential benefits of BCI within the industrial processes have been investigated in several works [6]. The aim is to facilitate the work of machine operators and limit risks. Lately, there has been enormous interest in BCI technology, as evidenced by the financing of large-scale projects focused on the advancement of the brain-machine interface, for example, Neuralink and neuronable [7,8], and an estimate of investments in this area put said technology on the market for a value of more than US $5.8 billion in 2020 [8].

Despite the above, BCI remains an open problem due to the complexity of the acquisition and processing of EEG signals. Regarding the acquisition of signals for use in BCI, there are several ways to measure activity in the brain. This task is generally divided into two categories: invasive and noninvasive [9]. Some invasive techniques include electrocorticography and single-unit action potentials. Most modern BCIs are based on noninvasive acquisition of electroencephalography (EEG) signals because they present less risk to the user. For noninvasive EEG acquisition, electrodes on the skin surface of the head are used [3]. However, the signals acquired noninvasively present extreme noise problems and other distortions, which makes their processing difficult. Additionally, the classification of multichannel EEG is an intensive and sophisticated task since those signals, even without noise, consist of dynamic and complex patterns of electric potential, exhibiting nonstationarity, high-dimensionality [3,10]. All this makes it difficult to implement BCI industrial cyber-physical systems, because the BCI systems, apart from presenting the problems mentioned above, have to respond with a minimum of latency to user signals in order to efficiently operate cyber-physical systems, this would require having sophisticated computing equipment on the industry floor to be able to process the user’s EEG, another alternative is to process the signals in the cloud; however, this could cause latency problems.

In this paper, we propose a fog-based architecture specific to managing the aspects of cyber-physical systems controlled through BCI. The proposed architecture aims to provide advanced connectivity, ensuring real-time response, accurate data acquisition from the physical layer, and information feedback from the cloud services through intermediate fog nodes. This will allow intelligent data management, collect analytics, and real-time computational capability. Thus, the contribution of this paper is twofold:

- It is proposed a new scheme for decoding EEG for use in BCI of cyber-physical systems.
- A new fog computing architecture for working with BCI control is developed in a distributed environment.

The subsequent sections of this paper are structured as follows: Section 2 provides an introduction to fog computing and BCI. Section 3, describes the architecture developed in this study. Section 4 presents the results obtained, and finally, Section 5, discusses the results.

## 2. Background

**Figure 1:**
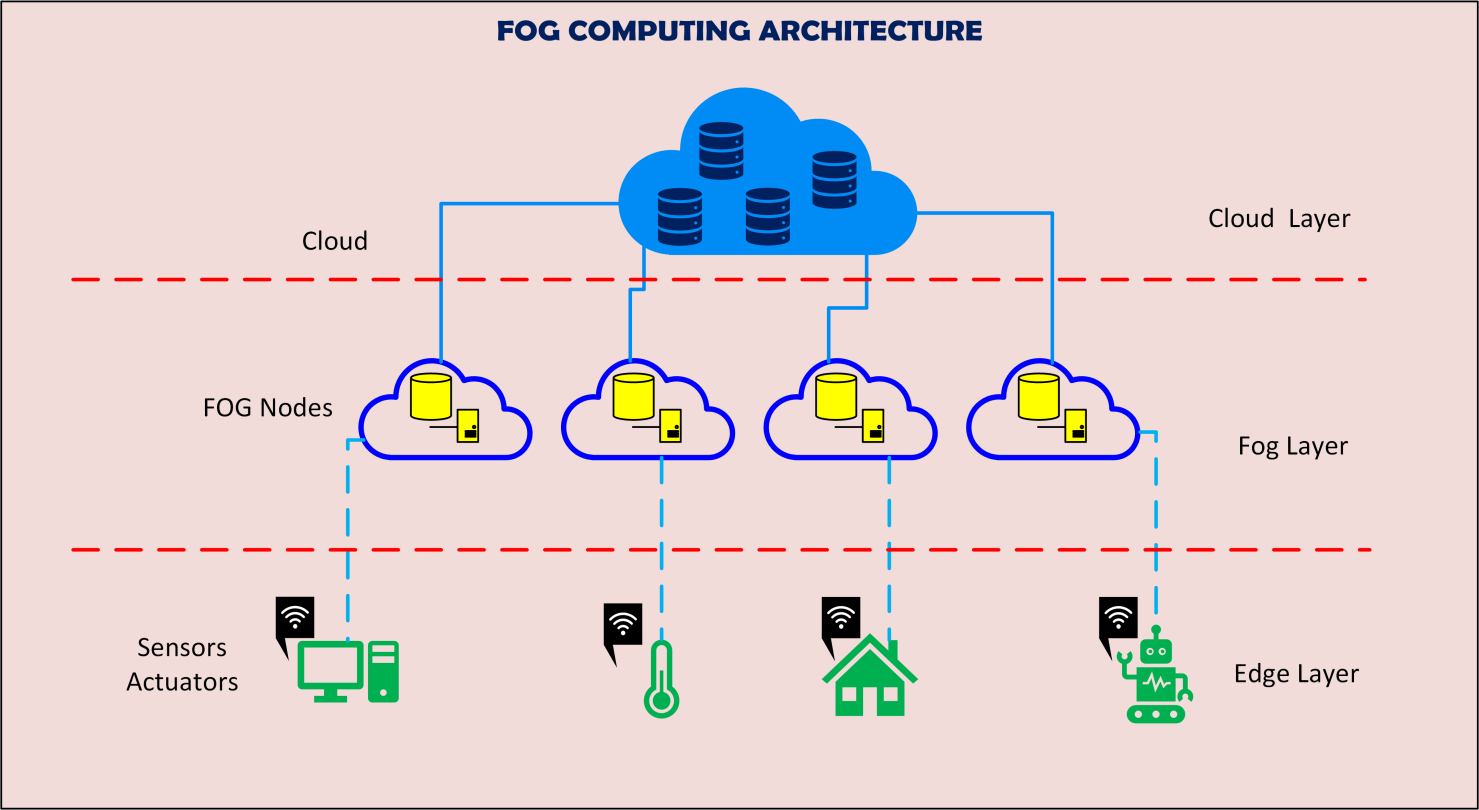
Overall architecture, fog nodes help to process and filter the data generated by the sensors, and the cloud server executes high level decisions pertaining to the general processing.

Fog computing extends the cloud computing paradigm whereby the data generated by devices at the edge are not uploaded directly to the cloud, but is first preprocessed in smaller decentralized fog nodes. This new layer functions in the middle between the edge layer (sensors and/or actuators) and the cloud layer, this is illustrated in Figure 1.

In most cases, these data must be processed by data analysis applications to gain valuable insights that inform decision-making about the monitored process. This processing can consume a lot of resources and overload cloud servers, making fog nodes crucial for ensuring seamless data transfer and mitigating traffic congestion in cloud servers, ultimately reducing latency.

Fog computing unlike cloud computing, is composed of a large set of closely distributed nodes with task-specific nodes of moderate capacity, while the cloud operates with a few high-capacity data centers [11]. Some advantages that provide fog computing are listed below [12,13]:

- Improves latency-sensitive applications: cloud server providers’ placement is generally far with respect to the edge devices which could cause latency problems for example in healthcare networks or vehicle networks. Fog nodes are typically placed close to IoT devices, helping to decrease latency.
- Increases cloud scalability problem: As the demand expands for cloud services, increases in cost and complexity may become unbearable. Fog computing helps in reducing computation resources diminishing the burden of cost-effective cloud resources.
- Save bandwidth: For example, with an increase in the demand for cloud services because of an excess of raw data for a wireless network, the spectrum could become a limited spectral resource when it is time to meet the requested service requirements [14]. However, if much of the raw data that arrive is processed by the fog nodes, only important, less dense information reaches the cloud instead of a large amount of raw data.

Brain-Computer Interface (BCI) technology decodes the brain activity of human beings in order to recover lost functions and improve their physical and cognitive abilities by connecting to a device. These interfaces have a defined process that includes the acquisition of brain activity, processing, classification of brain activity, and finally, the application or analysis of the acquired information [15].

As mentioned in the previous section, one of the most widely used techniques for acquisition is the electroencephalogram (EEG). EEG is a non-invasive and relatively low-cost technique compared to neuro-imaging techniques. EEG signals are characterized by their high temporal resolution and rapid response to internal or external stimuli; however, the spatial resolution is low due to the number of electrodes and the space occupied by the scalp [16]. In addition, they are susceptible to multiple factors internal to the subject, such as biochemical, metabolic, circulatory, hormonal, neuro-electrical, and behavioral factors [17]. They are also susceptible to alterations due to external stimuli such as sound, lighting and electronic noise [18].

The first EEG studies were performed by Richard Caton around 1875; in 1929 the first experiments on humans were reported by Hans Berger; however, it was not until 1990 when the first applications of EEG in the area of bionics were performed by the U.S. Department of Defense [19].

Electroencephalographic signals are obtained from the excitatory postsynaptic potential, which is generated due to the exchange of neurotransmitters between the sending and receiving cell [20]. These signals are captured due to the potential difference between an active electrode placed on the scalp and a reference electrode, which are amplified [21]. EEG signals are displayed as temporal sine waves with respect to their amplitude which normally ranges from 0.5 to 100 microvolts and can be displayed based on their power spectrum which is divided into ranges called beta (>13 Hz), alpha (8 Hz–13 Hz), theta (4 Hz–8 Hz), delta (0.5 Hz–4 Hz) [22].

On the other hand, brain activity for BCIs is generated from two distinct paradigms, which can be elicited by external visual or auditory stimuli, and generated by internal stimuli such as the Motor Imagery (MI) paradigm [23]. MI is a technique in which the movement of a limb is imagined by modulating the frequency of the signal in the alpha and beta ranges, this modulation is known as event-related desynchronization (ERD) and event-related synchronization (ERS) [24]. The MI paradigm is performed for trained subjects such as athletes and untrained subjects [25].

Recent MI studies have shown that the brain regions that provide the greatest informational weight in the classification of EEG signals are the central region, which is responsible for motor activity, corresponding to the C3, C4, and Cz regions, mainly according to the 10–20 electrode placement system [26]. However, the study of [27], shows that the frontal region is the one that provides the most information for signal classification.

On the other hand, BCI applications based on MI are classified into two main groups, medical and non-medical applications. Medical applications focus on restoring motor functions lost during spinal cord injury through stimulation for brain regeneration and plasticity [28], another medical application is the substitution of movement through BCI for control or communication, such is the case of [29]. On the other hand, non-medical applications are used to increase human capabilities and entertainment; such as the case of control of robotic arms and exoskeletons [30], control of ground vehicles [31], drones [32], and games in virtual environments [33].

After the acquisition of the electroencephalographic signals, the processing stage follows. This stage varies according to the study design and ranges from the selection of acquisition channels [34], signal filtering, and elimination of artifacts and noise, since EEG signals have a low signal-to-noise ratio (SNR) [35].

One of the most commonly used algorithms at this stage is independent component analysis (ICA), for example in the [36] study they use ICA to optimize the characterization of subject-independent MI signals, based on the ERD/ERS paradigm, improving the accuracy between 6.9% and 7.9%. Another study is [37] which proposes a method to extract features from low-quality EEG signals in combination with ICA.

After processing, the next step is feature extraction, which can be spatial, the most commonly used technique being Common Spatial Patterns (CSP) [38]. Temporal characteristics which are generally statistical techniques [39]. And spectral characteristics such as FFT, PSD, DWT [40,41,42].

However, during the processing and feature extraction stage, there is a high possibility of losing important information. That is why recent studies have shown that neural networks have the ability to learn complex features from high-dimensionality temporal data which can be even in raw [43].

During the classification stage, several classical machine learning algorithms such as Random Forest (RF) [44], Decision Tree (DT) [45], Support Vector Machine (SVM) [46], as well as deep learning algorithms in which hybrid neural networks stand out, the most common is the hybridization of convolutional neural networks (CNN) and recurrent neural networks such as Long short term memory (LSTM) [47,48]. They have been used in EEG signal classification methods for BCI to increase accuracy. However, it is necessary to apply BCI to the real world, so this paper proposes to decrease the latency in classification using fog computing.

## 3. Materials and Methods

In this section, we discuss the materials and methods proposed for this research.

### 3.1. Fog Computing for BCI Based System

**Figure 2:**
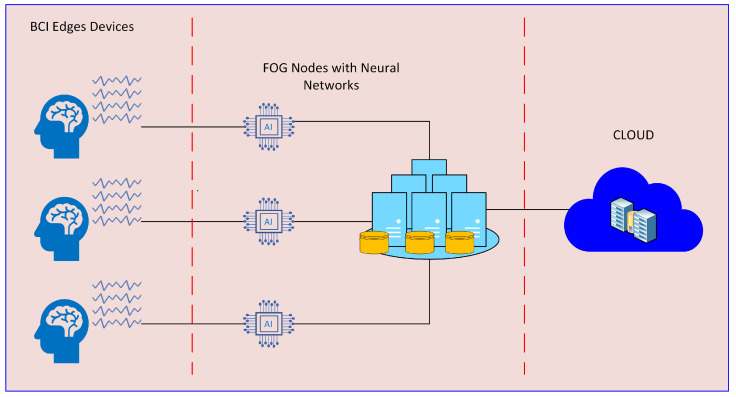
Overall Fog BCI architecture, fog nodes help to process and filter the EEG data acquired from sensors at the scalp, the cloud server executes high level decisions pertaining the general processing.

The proposed architecture is illustrated in Figure 2. This architecture proposes the use of MI BCI in an industrial setting to control a cyber-physical system using an IoT device consisting of a Raspberry Pi and an EEG headset for acquiring MI EEG data. The acquired data are transmitted to a fog node which filters the raw data and uses a neural network to encode the data into commands. These commands are then sent to the cloud for validation, analysis and control.

### 3.2. BCI System Architecture

**Figure 3:**
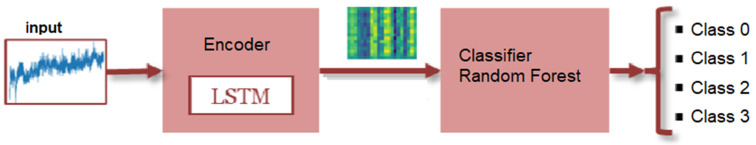
Proposed machine learning model for EEG classification. In this study, the EEG signal will be classified into one of the following four classes: 0-movement of the left foot, 1-left hand (LH), 2-state of relaxation (RX), and 3-math activity (MA).

The illustration in Figure 3 outlines the machine learning model being proposed for categorizing EEG signals into commands. The input of the model is a raw EEG signal, x∈X, where X represents the signals in the database. Each signal *x* is then transformed into a latent representation, F, through encoding. This new representation is expected to contain relevant information that the classification algorithm, a random forest, uses to determine the command that the input signal corresponds to.

The latent space in this case is an image or matrix space. The concept of transforming signals into images has been used in prior works, such as converting an ECG into 32×32 binary images in [49], and utilizing a Gramian Angular Summation Field to transform traffic time-series data into images in [50]. In this work, we propose using a deep learning encoder to accomplish this task.

**Figure 4:**
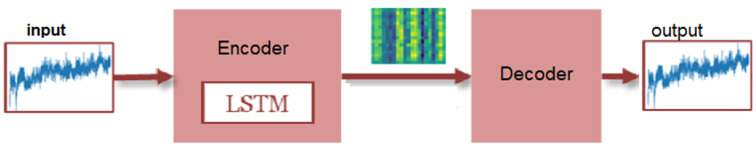
Autoencoder for encoding an EEG signal to an image.

For this an encoder was trained using an autoencoder with the signals in X of the database, the architecture is shown in Figure 4.

The input and output of the autoencoder are the same signal, such networks consist of two parts the encoder, $f_{enc}:X\to F$, and a decoder part, $f_{dec}:F\to X$. The encoder takes signals from X and maps them to a latent space F that is subsequently transformed again into the signal space by the decoder. The training of the autoencoder consists of obtaining $f_{enc},f_{dec}$ such that

(1) $$f_{enc},f_{dec}=\underset{f_{enc},f_{dec}}{arg min}||X-\left(f_{dec}\circ f_{enc}\right)X{\left|\right|}^{2}$$

In this study it is proposed to use a convolutional LSTM layer for the encoder, since convolutional LSTMs are designed to work with sequential data, they are well-suited for handling the sequential nature of EEG signals, which represent temporal activity in the brain. Their capability in managing the brain’s electrical activity over time and their adaptability for learning long-term dependencies make them effective in capturing temporal patterns within EEG signals. Furthermore, the capacity of LSTMs to selectively retain or discard information across extended sequences is of great help, especially for EEG signals characterized by intricate patterns distributed over time. Additionally, in the case of EEG signals, where relevant features may not be immediately obvious, LSTMs can automatically learn and extract features from the input data, eliminating the need for manual feature engineering. The proposed architecture is described below. The encoder used involves a convolutional LSTM layer with 20 filters and a kernel size of two. The layer is then reshaped to a 3×20 dimensional output tensor, followed by a convolutional transpose layer consisting of a single filter of size 14×2, resulting in an output tensor (image) of size 16×21.

The decoder is composed of a flatten operator as its initial step, followed by a long short-term memory layer that contains two neurons, then a convolutional layer with three filters of size four. It also includes two convolutional transpose layers that have three filters of size three, and three convolutional layers with seven filters of sizes 90, 90, and 11, respectively. The final step in the decoder is a convolutional layer with four filters of size ten. It is important to note that the autoencoder is only utilized during training and is not part of the final implementation.

As shown in Figure 3 only the encoder part was used in the final model. In this way, the input signal is transformed into an image. It is expected that this image contains relevant characteristics for its classification. This classification is carried out by a random forest. The Algorithm 1, comprises the classification module that takes an EEG signal as input. This signal is then processed through the encoder and classified using a random forest. Finally, the module returns the result of the classification.
**Algorithm 1** Classify signal**Input:** EEG signal x∈X
**Output:** C {class of the signal}
$h=f_{enc}\left(x\right)$ {signal in latent space}
C=RF(h) {classification by RF}
return*C*


### 3.3. Data Used to Train the Network

For training the network, the MI EEG database from [51] was used, where the data acquisition was carried out using a MUSE^™^ device, that is, a headset that detects EEG signals. Because it is non-invasive, it is harmless to acquire signals from the brain; therefore, this headband allows the measurement of brain activity in real-time without affecting the human being.

**Figure 5:**
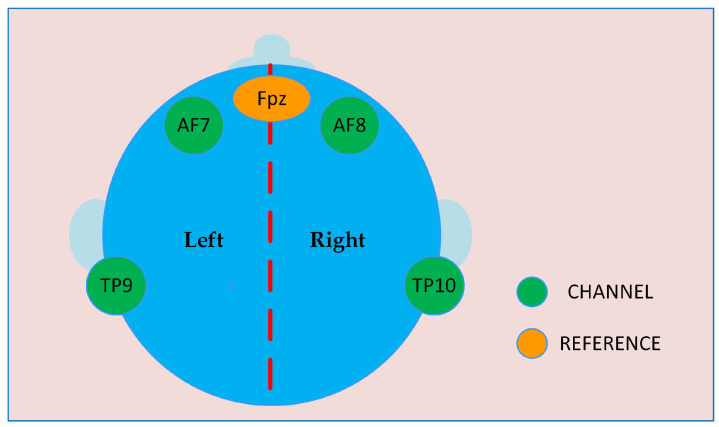
Channel locations used by the EEG acquisition device.

Specifically, the EEG acquisition device works with four channels. These channels are used by bipolar gold electrodes, which are placed in the area of the sensorimotor cortex at locations AF7, AF8, TP9, and TP10, of the 10–20 system, as shown in Figure 5. For this database, subjects were instructed not to physically perform any kind of movement, instead, the subject simulated a given action using Motor imagery (MI). As a result, an amount of 200 records distributed among the following activities was obtained through MI: movement of the left foot (LF), left hand (LH), state of relaxation (RX), and math activity (MA).

### 3.4. Fog Integration

Consider the following scenario, a machine operated by a person using BCI on an industrial plant floor. The person wears a headband to gather EEG signals which are used to control the machine in real-time. This scenario is repeated by each operator and both the headbands and machines could differ from one another.

**Figure 6:**
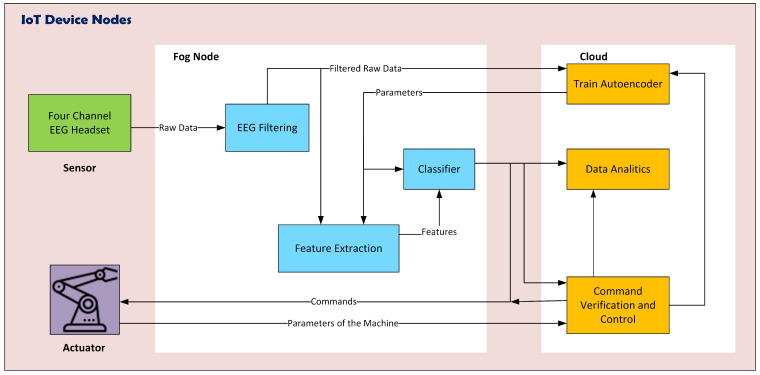
Fog computing module.

It would be challenging to entirely build the scenario mentioned above using only cloud computing as real-time processing of EEG data requires processing units to be as close to the data source as possible to reduce latency and network congestion. This limits the use of cloud computing for application management and global coverage. However, fog computing is a suitable solution for these types of issues where proximity and global coverage are required [52]. This is why the use of fog computing is critical for the objectives of this paper as it is expected to lead to low latency and reduce the need for constant cloud connectivity. In Figure 6, we illustrate the proposed fog computing solution, consisting of three layers specific to the environment of this work, adapting a common architecture for fog applications. The IoT layer includes EEG acquisition devices as sensors and a cyber-physical system for control, serving as the actuator. The fog layer comprises dedicated fog nodes, each incorporating EEG signal processing mechanisms such as the filtering module, feature extraction, and the classifier. The fog node has the capability to directly control the cyber-physical system or transmit the classifier’s output to the cloud. The cloud is equipped with three services: one for retraining the classifier to obtain new parameters, another for statistical data analysis, and a command module. The command module not only controls the cyber-physical system based on the classifier’s output but also receives data from the cyber-physical system for verification purposes. The fog layer and the cloud layer are described in more detail below.

#### 3.4.1. Fog Nodes

The proposed solution involves incorporating intelligence into fog computing nodes. The fog node consists of three main blocks: filtering, feature extraction, and machine learning (ML) model for classification. The raw data from four EEG channels are received as input from the IoT layer and is filtered before being passed to the feature extraction block, which is made up of a previously trained encoder. The resulting features are then classified by the ML model, which consists of a previously trained random forest classifier. The classifier determines the EEG signal’s command. The feature extraction modules and classifiers can also be retrained from the cloud by updating their parameters, giving the nodes more flexibility.

#### 3.4.2. Cloud

The cloud provides several services with high computing capacity, the input data from the fog layer, are mainly commands and other parameters pertaining to the machine. The cloud hosts a data analytics module; command verification and control and ML trainer. The data analytics module performs the systematic computational analysis of data and statistics of machine usage and performance. This module consists mainly of diagnostic analytics, analysis of the current state of operations, predictive analytics, and command analytics. The command verification and control module verifies commands from the classifier and issues further commands to operate the machine. In addition, it instructs the ML trainer module if it is necessary to retrain the ML models, in case of operator change, or repetitive anomalous commands. The ML trainer contains the ML models in order to retrain them if necessary. Because the computational cost of training is high, this process is conducted in the cloud. At the output of this module are the parameters of the trained models.

## 4. Results

This section presents the results of the performance evaluation of the classifier. In this section, the performance evaluation results of the classifier are presented. The database discussed in Section 3.3 was used, with a partition of the data into 80% for training and 20% for testing.

### 4.1. Encoder of EEG

**Figure 7:**
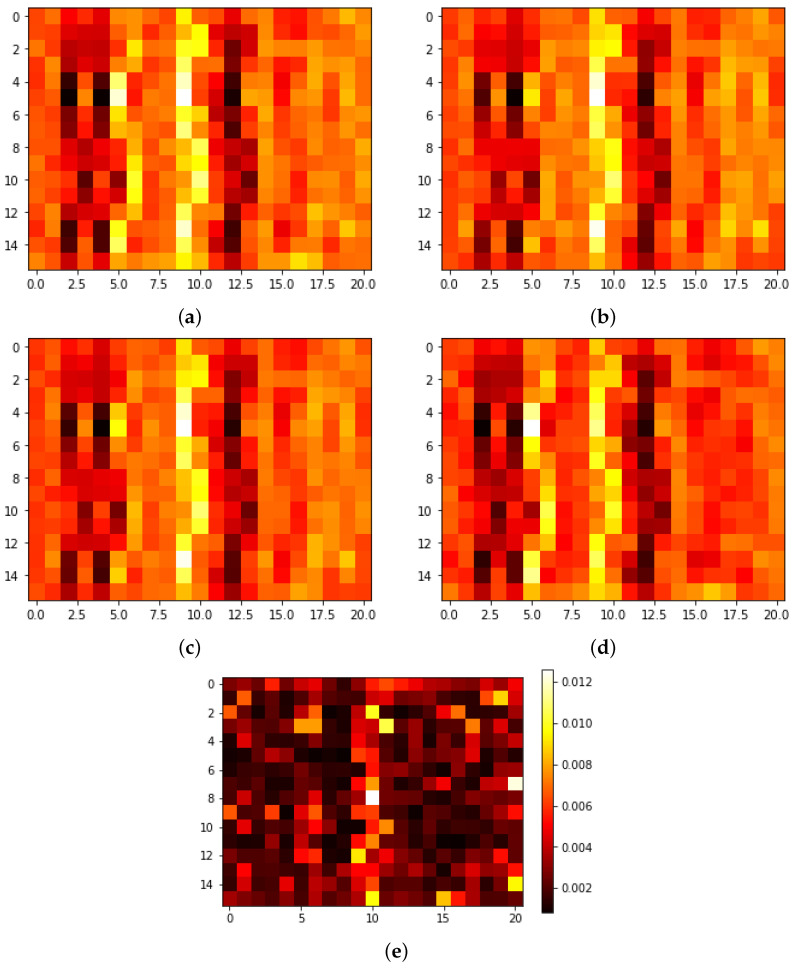
Average images produced by the encoder for each class. (**a**) LF, (**b**) LH, (**c**) RX, (**d**) MA, and (**e**) importance of each pixel according to the RF.

Here, we investigated the data produced by the encoder. As explained in the Section 3.2 the encoder transforms an EEG signal into a 16×21 image. Figure 7a–d, shows the average images for each class. Figure 7e shows a visual representation of the significance of each pixel assigned by the RF classifier, with the significance indicated by color. In this context, the assumption is made that every pixel represents a feature. The significance of each feature is determined using the Mean Decrease in Impurity or Gini Importance method, as assessed by the random forest [53].

**Figure 8:**
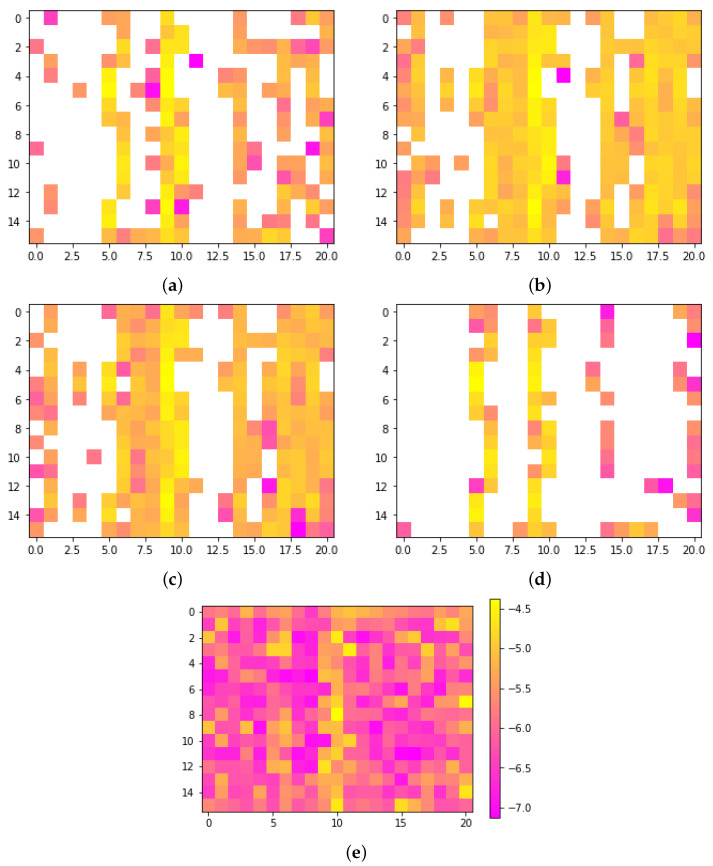
Log average images produced by the encoder for each class. (**a**) LF, (**b**) LH, (**c**) RX, (**d**) MA, and (**e**) importance of each pixel according to the RF.

In Figure 7, it is evident that the central vertical band is typically the most significant. However, the average images do not reveal a clear pattern distinguishing the classes. On the other hand, when the logarithm of the positive pixels of each class is taken, Figure 8, a trend is observed. The LH and RX classes, depicted in Figure 8b,c, exhibit distinct characteristics. Notably, these classes reveal vertical clusters prominently positioned at the center and right of the images, displaying a substantial concentration of pixels. In contrast, the LF and MA classes display pixel clusters with lower density. It is important to qualitatively observe that the distribution pattern of clusters in the LH and RX classes bears greater similarity to each other, while there is also some similarity between the LF and MA classes. We anticipated that the LF and LH classes would be similar since both classes have to do with extremities of the body, but this was not the case, instead, the LH and RX classes are more are more similar to each other, as are the LF and MA classes. The reason behind the apparent similarity between the LH and RX classes in the results could be attributed to the fact that hand movements are frequently performed in daily life and do not require much mental effort, as is the case with RX. In contrast, moving the foot (LF) is not a common task and requires more focus from the user, similar to MA.

### 4.2. Classifier Performance

For the selection of the classifier, different ML algorithms were evaluated using the encoder output: Random forest (RF) with 250 estimators; support vector machines (SVC), with C=1.0 and rbf kernel; Gradient Boosting (GB) with 159 estimators, maximum depth of four; decision tree (DT) with maximum depth of the tree and gini for split quality. All algorithms were implemented using the scikit-learn library [54], also, library default values were used for additional algorithm hyper-parameters.

**Figure 9:**
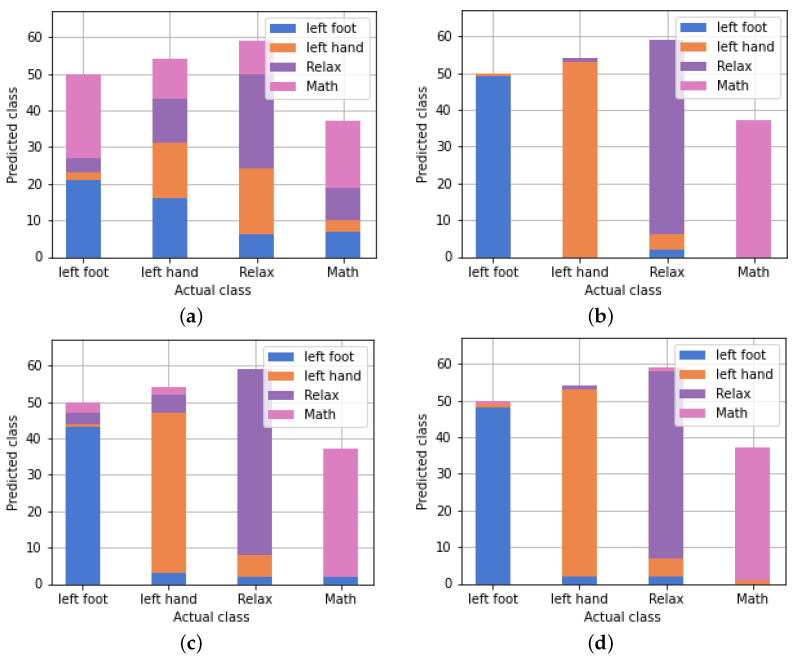
Performance of the classifiers. The proportion of samples that were assigned to a given class is shown graphically for each class. (**a**) SVM, (**b**) Random Forest, (**c**) Decision Tree, and (**d**) Gradient Boosting.

The results are presented in Table 1 and show that the Random Forest (RF) classifier performed the best with an accuracy (acc) of 0.96, followed by the Gradient Boosting (GB) classifier with an accuracy of 0.93. RF also achieved the best evaluation in terms of precision, recall, and f1-score across all classes. Figure 9 depicts the performance of each classifier, with each bar representing a class and displaying the percentage assigned to the classes determined by the algorithm.

Although the highest performance of the algorithms evaluated does not achieve a perfect accuracy of 100%, the system can still be used to control types of cyber-physical systems that are more forgiving towards occasional errors. For instance, it can be utilized to control conveyor belts, quality control lights for products, and semi-automated cyber-physical systems that only require the material to be placed at the start of the cycle and then the machine delivered once it is finished, such as the case for pre-formed tubes or plastics.

For instance, consider the use of the system in a quality control lights for products scenario, where an operator checks the product and raises the red light alarm if they spot any damage. Even if the system triggers a false alarm, it would not have a significant impact on the business, as another operator would simply verify that the product is not damaged. In the event that the BCI system fails to detect a command and an alarm is not raised, the operator will have another opportunity to try again. The likelihood of the BCI system failing to detect the command twice is low, for example, with the acc of the RF of 0.96, the probability is 0.04×0.04. The aim of the proposed system is to enable the integration of disabled individuals into the workforce, improving their self-esteem and keeping them occupied with a sense of purpose, thereby promoting self-sufficiency, person-centeredness, and community integration [55].

### 4.3. Fog Architecture Performance

**Figure 10:**
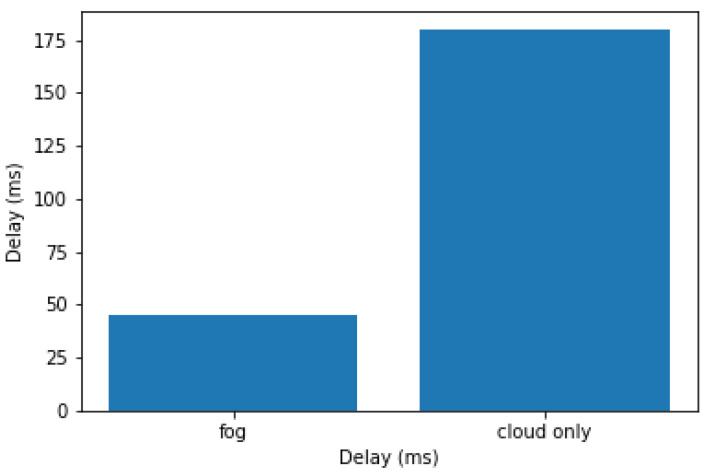
Average latency.

We have simulated a fog computing environment using YAFS (Yet Another Fog Simulator) a fog computing simulator for analysis of designs and performance of deployment of applications [56]. Two simulations were carried out. The first simulation used a fog node for processing and classifying the EEG signal, while the second used only a cloud node. The results of the simulations are shown in Figure 10 where you can see how the fog node greatly reduces latency.

## 5. Discussion

This paper introduces a novel BCI system that utilizes fog computing, showcasing a design that integrates EEG acquisition devices, fog nodes, cloud nodes, and cyber-physical systems. The designed architecture consists of processing the EEG signal through a convolutional LSTM, combining an autoencoder and a random forest for signal classification into commands for controlling a cyber-physical system. The proposed architecture exhibits superior performance, achieving a precision of 0.96, outperforming alternative classifiers. The utilization of a Random Forest-based architecture not only enhances precision but is also easy to implement and imposes a low computational burden, making it suitable for deployment on embedded systems hosting the fog node.

In evaluating the system’s performance through simulation, we observed that the integration of fog-cloud computing resulted in significantly reduced latency compared to a system relying solely on cloud computing. This achievement emphasizes the effectiveness of our approach to employing fog computing with machine learning algorithms to convert EEG signals into commands for cyber-physical systems, ultimately enhancing responsiveness and performance.

While the utilization of machine learning and fog computing for Brain-Computer Interfaces in industrial cyber-physical systems holds significant potential, it is essential to address possible limitations and challenges to ensure the system’s reliability, security, and adaptability in real-world scenarios. Future work should consider the following aspects: testing with a larger pool of subjects, as EEG signals exhibit high variability due to factors such as individual differences, mental states, and environmental conditions; enhancing the robustness of the filtering module to increase resistance to disturbances such as noise and artifacts; addressing security and privacy concerns, given the critical nature of industrial systems, and developing modules with robust security measures to safeguard sensitive EEG data and improving interoperability with existing equipment, particularly in industrial settings where legacy equipment is prevalent. Finally, it is necessary to devise techniques to address the scalability of the system, especially as the amount of EEG data collected and used for training the machine learning model increases. The system’s capacity to handle larger datasets while maintaining performance is key in industrial environments. With the expansion of the user base, the data volume may grow significantly, necessitating scalable solutions.

## Figures and Tables

**Table 1:** Performance metrics results.

	Precision				Recall				f1-Score				Accuracy
	LF	LH	RX	MA	LF	LH	RX	MA	LF	LH	RX	MA	
SVM	0.42	0.39	0.51	0.30	0.42	0.28	0.44	0.49	0.42	0.33	0.47	0.37	0.40
RF	0.96	0.91	0.98	1.00	0.98	0.98	0.90	1.00	0.97	0.95	0.94	1.00	0.96
DT	0.88	0.81	0.91	0.82	0.88	0.81	0.86	0.89	0.88	0.81	0.89	0.86	0.86
GB	0.92	0.88	0.98	0.95	0.96	0.94	0.86	0.97	0.94	0.91	0.92	0.96	0.93

## Data Availability

The data that support the findings of this study are available upon reasonable request.

## Abbreviations

The following abbreviations are used in this manuscript:

BCI	Brain computer interface
MI	Motor Imagery
EEG	Electroencephalogram
LF	Left foot
LH	Lesf hand
RX	Relaxation state
MA	Matemathical activity
RF	Random forest
DT	Desition tree
SVM	Support vector machine
GB	Gradient Boosting
